# Draft genome sequences of *Pseudomonas* strains zfem001–005 isolated from the intestine of larval zebrafish *Danio rerio*

**DOI:** 10.1128/mra.00934-23

**Published:** 2024-02-20

**Authors:** Sabona B. Simbassa, Justin Clark, Keiko Salazar, Anthony Maresso, Anne- Marie Krachler

**Affiliations:** 1 Department of Microbiology and Molecular Genetics, The University of Texas Health Science Center at Houston, Houston, Texas, USA; 2 Microbiology and Infectious Diseases Program, University of Texas MD Anderson Cancer Center UTHealth Houston Graduate School of Biomedical Sciences, Houston, Texas, USA; 3 Department of Molecular Virology and Microbiology, Baylor College of Medicine, Houston, Texas, USA; 4 Tailored Antibacterials and Innovative Laboratories for Phage (Φ) Research (TAILΦR), Baylor College of Medicine, Houston, Texas, USA; Loyola University Chicago, Chicago, Illinois, USA

**Keywords:** *Pseudomonas*, nonhuman microbiome, zebrafish, intestinal colonization, *Danio rerio*

## Abstract

Here, we report the draft genome sequences of *Pseudomonas* strains zfem001–005, five isolates from the intestinal microbiota of healthy larval zebrafish *Danio rerio* at a developmental age of 7 days post fertilization. The isolates have been identified as *Pseudomonas sediminis*, *Pseudomonas japonica*, *Pseudomonas otitidis*, *Pseudomonas sichuanensis*, and *Pseudomonas tohonis*, respectively.

## ANNOUNCEMENT

Larval zebrafish are increasingly used as a vertebrate model for intestinal host–microbe interactions due to their genetic tractability, optical transparency, and a physiology that shares many similarities with mammalian models (1). The endogenous microbiota and its interactions with larval and adult zebrafish have been extensively characterized (2, 3). These studies have shown that, although larval zebrafish are colonized early during development and form a stable gut microbiome, the composition of the microbiome varies between different facilities (4). In order to provide a resource for colonization studies on larval zebrafish, we have isolated five of the most abundant isolates from the intestinal tissues of larval zebrafish derived from the Center for Laboratory Animal Medicine and Care aquatics facility at UTHealth Science Center at Houston at 7 days post fertilization (dpf) and sequenced their genomes.

**Fig 1:**
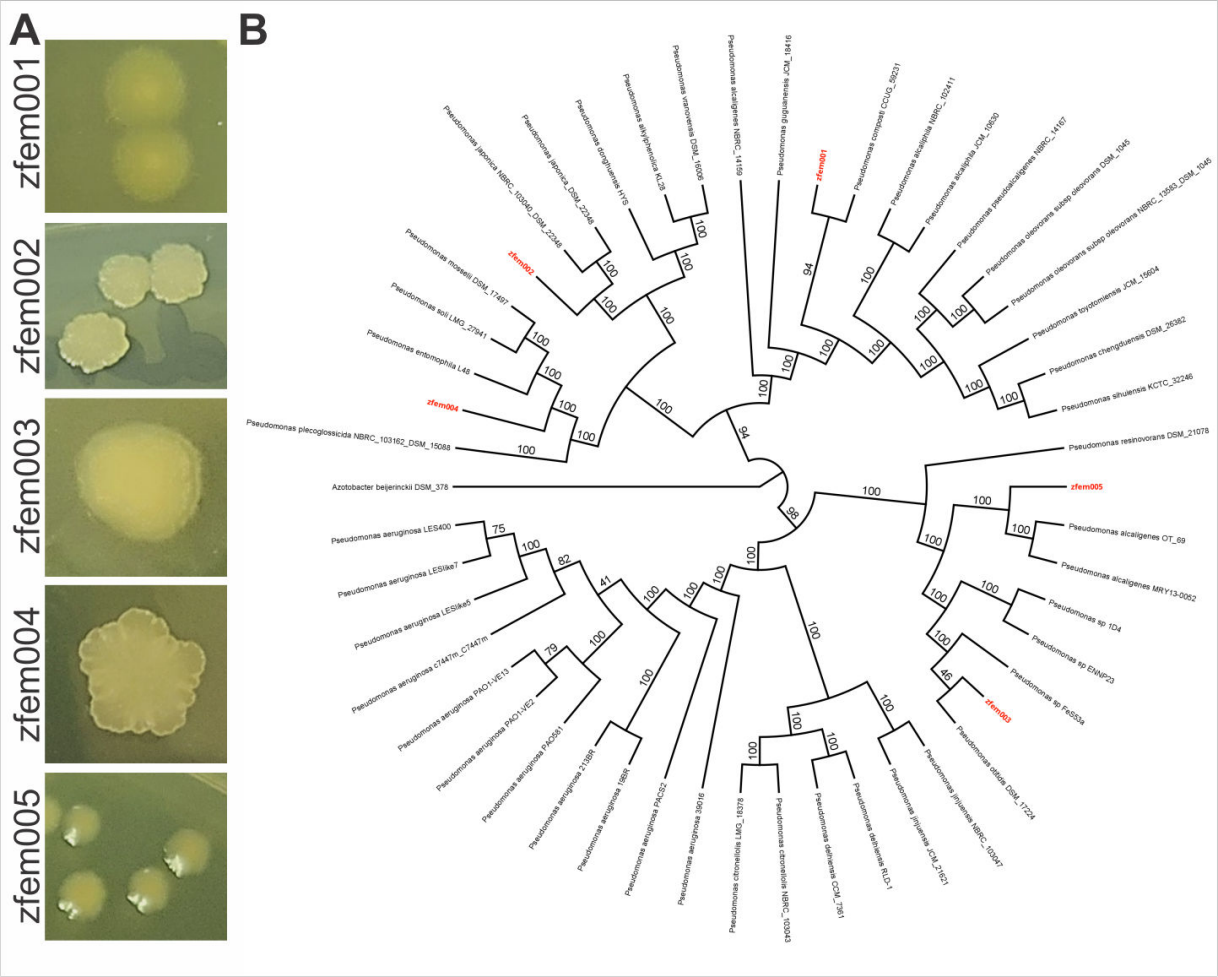
(**A**) Colony morphology of *Pseudomonas* isolates zfem001–005 grown on LB agar. (**B**) Phylogenetic tree was generated using autoMLST in *de novo* mode with concatenated alignments and 1,000 ultrafast bootstrap replicates. Branch labels show percent consensus bootstrap support. Tree is rooted in *Azobacter beijerinckii* DSM 378 (accession number NZ_FOFJ00000000.1).

At 7 dpf, larval zebrafish were euthanized, and intestinal tissues were homogenized, diluted, and plated on lysogeny broth (LB) agar as described previously (5). Following growth on LB agar at 37°C for 20 h, individual colonies were picked and streak purified twice on LB agar, with an incubation at 37°C for 20 h each time (colony morphologies; see Fig. 1A). Prior to DNA isolation, individual colonies were used to inoculate LB, and cultures were grown at 37°C for 20 h, shaking at 185 rpm.

Genomic DNA was extracted from all five isolates using the Omega E.Z.N.A. Bacterial DNA Kit and was sent to Novogene for sequencing on the Illumina HiSeq 4000 platform. Raw reads were trimmed to a quality score of 30, and reads under 50 bp were filtered out using BBDuk (v.38.84) with default parameters (6). Trimmed reads were subsampled using Geneious Assembler to bring assembled coverage depth to between 40× and 80× and then assembled using Geneious Assembler in Geneious Prime (v.2022.1.1) (https://www.geneious.com), with medium-low sensitivity settings. Contigs that were larger than 1,000 bp and consistent with the largest contigs in terms of coverage were extracted and used as a reference to validate all reads by mapping all reads to the reference in Geneious Assembler with settings to detect variants of any size. For detailed assembly statistics, see Table 1. Assemblies were then annotated using the National Center for Biotechnology Information (NCBI) Prokaryotic Genome Annotation Pipeline (v.6.6) (7) and default parameters. BLAST was used to search assemblies against the NCBI 16S ribosomal RNA sequence database to find closely related strains (8, 9). A tree was created using autoMLST in *de novo* mode with a concatenated alignment and 1,000 IQ-TREE Ultrafast Bootstrap replicates (10). The displayed taxa were chosen manually from the strains in the *Pseudomonas* genus that were among the 50 closest species identified by autoMLST. The phylogenetic tree (Fig. 1B) was plotted using Geneious Tree Viewer and rooted in *Azobacter beijerinckii* DSM 378 (accession number NZ_FOFJ00000000.1). Species designations (Table 1) were determined using PubMLST (v.1) (https://pubmlst.org/) (11).

**TABLE 1:** *Pseudomonas* isolates zfem001–005 properties and assembly statistics

Isolate ID	PubMLST species ID	Genome size (bp)	GC content (%)	Number of contigs	Number of reads	Assembly stats	N50 length (bp)
*Pseudomona*s zfem001	*Pseudomonas sediminis*	5,150,040	62.4	12	10.8M	Assembled using 2,943,983 paired hits and 1,740 unpaired hits	469,972
*Pseudomonas* zfem002	*Pseudomonas japonica*	6,799,339	64.3	57	12.6M	Assembled using 2,854,410 paired hits and 3,098 unpaired hits	202,513
*Pseudomonas* zfem003	*Pseudomonas otitidis*	6,825,556	67.1	25	13.8M	Assembled using 3,115,034 paired hits and 3,438 unpaired hits	535,079
*Pseudomonas* zfem004	*Pseudomonas sichuanensis*	5,828,281	64	50	13.8M	Assembled using 2,453,113 paired hits and 1,857 unpaired hits	201,843
*Pseudomonas* zfem005	*Pseudomonas tohonis*	6,927,531	66.2	27	10.8M	Assembled using 1,974,192 paired hits and 838 unpaired hits	423,767

## Data Availability

The draft genome sequences of five *Pseudomonas* strains isolated from larval zebrafish have been deposited at GenBank under accession numbers SRX22040690 (zfem001 chromosome), SRX22040691 (zfem002), SRX22040692 (zfem003), SRX22040693 (zfem004), and SRX22040694 (zfem005)
